# Why ICF? advantages of ICF in the clinical practice with regard to the medical care of people with mental health problems and intellectual disabilities

**DOI:** 10.1192/j.eurpsy.2021.199

**Published:** 2021-08-13

**Authors:** M.-M. Theil

**Affiliations:** Praxis Dr. Med. M.-m. Theil, Sanamens Praxisgemeinschaft, Helmstedt, Germany

**Keywords:** ICF, Intellectual Disabilities, mental health, Social Medicine

## Abstract

**Introduction:**

The International Classification of Functioning, Disability and Health (ICF) provides a framework rooted in patient-centered care and the biopsychosocial model that facilitates a comprehensive description of a person’s health and their level of societal participation. The importance of the ICF for assessing the needs of individuals with mental health problems (MHP) and intellectual disabilities (ID) is growing, especially in the social medicine.

**Objective:**

To describe the benefits and limitations of the ICF in clinical practice, pertaining to the assessment of healthcare needs and societal participation in persons with MHP and ID.

**Method:**

Comprehensive literature search in medical databases using the Keywords: ICF, mental health, intellectual disabilities, social and occupational participation.

**Results:**

ICF-based instruments such as the Mini-ICF-APP, with which impairments and competencies in social and occupational participation can be described, are playing an increasingly important role in healthcare and rehabilitation. In Germany, for example, in accordance with the Federal Participation Act, the entitlement to disability support benefits is assessed using ICF-based instruments, which therefore play a decisive role in social medical care.

**Conclusion:**

The functional descriptions of the ICF provide the opportunity for a standardized, yet individualized assessment of medical needs, general health and societal participation, thus facilitating the provision of a comprehensive package of care and support for people with disabilities. ICF-Core Sets and the Mini-ICF-APP are effective tools to describe level of function. It would be clinically valuable to further develop these instruments for use in persons with ID and MHP in the field of social medicine.

**Disclosure:**

No significant relationships.

